# EDM-based analysis of Fe-based shape memory alloys using Cu-W electrodes with multiple output optimization and microstructural validation

**DOI:** 10.1038/s41598-025-14013-z

**Published:** 2025-08-02

**Authors:** Ranjit Singh, Sambit Satpathy, Dhirendra Kumar Shukla, Ravi Pratap Singh, Rajeev Trehan, Jibitesh Kumar Panda, Biplab Bhattacharjee

**Affiliations:** 1 Department of Industrial and Production Engineering, NIT Jalandhar, Jalandhar, 144027 Panjab India; 2 CSE Department, Galgotias College of Engineering and Technology, Greater Noida, Uttar Pradesh, 201306 New Delhi India; 3 CSE Department, Galgotias University, Greater Noida, 203201 Uttar Pradesh India; 4 Department of Mechatronics, Manipal Institute of Technology, Manipal Academy of Higher Education, Manipal, 576104 Karnataka India; 5 Department of Mechanical Engineering, Faculty of Engineering and Technology, SRM Institute of Science and Technology, Ramapuram, Chennai, 600089 India

**Keywords:** EDM, WOW, WOTE, RSM, CCD, SEM, SMA, Desirability, Materials for energy and catalysis, Nanoscale materials, Structural materials, Energy science and technology, Engineering, Materials science

## Abstract

Shape Memory Alloys (SMAs) are pivotal in diverse industrial applications due to their exceptional properties, including actuation, biocompatibility, and adaptability in aerospace, biomedical, and military domains. However, their complex machinability often leads to high costs and suboptimal surface quality when processed using traditional methods. Using Response Surface Methodology (RSM) with a Central Composite Design (CCD), this study evaluated the effects of input parameters, including pulse on time (T_on_), pulse off time (T_off_), peak current (Ip), and gap voltage (GV), on material wear responses during Electrical Discharge Machining (EDM). Fe-based Shape Memory Alloys (SMAs) were machined using a Cu-tungsten electrode to investigate the wear characteristics of both workpieces and tool electrodes. Results revealed that Workpiece Material Removal Rate (WOW) ranged from 11.30 to 65.17 mm³/min, and Tool Wear Rate (WOTE) varied from 0.0062 to 0.01127 g/min. Scanning Electron Microscopy (SEM) of machined surfaces showcased craters, micro-cracks, and recast layers, elucidating the correlation between process parameters and surface integrity. Multi-objective optimization using the desirability approach identified optimal conditions for balancing machining efficiency and surface quality. This research provides a comprehensive understanding of the EDM process for Fe-based SMAs, paving the way for improved machinability and expanded industrial applications.

## Introduction

Smart materials are increasingly demanded for exceptional properties and applications. These materials, with their inherent adaptability and unique characteristics, are driving the evolution of intelligent systems that integrate sensors, microcontrollers, and actuators. Among these, shape memory alloys (SMAs)—a subset of shape memory materials (SMMs)—are particularly notable for their ability to “remember” and revert to their original shape upon exposure to specific magnetic or thermal stimuli, a phenomenon referred to as the shape memory effect^1–4^. Since their introduction as “smart alloys,” SMAs have been developed into various forms, such as solids, films, and foams, and are predominantly based on NiTi, Cu, and Fe alloy systems. These alloys are increasingly utilized in industries ranging from robotics to biomedicine, owing to their high versatility and functional efficiency. However, the machinability challenges associated with SMAs, due to their high toughness, ductility, and intricate geometrical requirements, have limited their broader industrial adoption^5–9^.

Despite SMAs’ outstanding and superior properties, they are not widely used since conventional procedures have a hard time treating them, leading to expensive processing costs and subpar surface quality. The importance of high-quality products is growing in this age of rapid technological advancement. Various state-of-the-art technical materials (superstructured alloys, SMAs, ceramics, composites, etc.) are produced by means of thermal, mechanical, and electrochemical energy in a number of complex production processes^10–12^. One example of innovative manufacturing practices is non-traditional machining processes. It is used to facilitate material machining by acting as a barrier between the tool and the workpiece. Electrical discharge machining (EDM) is highly effective for processing materials that are challenging to machine using conventional methods^13–16^. Its application in achieving superior machining precision, tackling complex geometries, and improving surface finish is becoming increasingly prevalent across industries^13–17^. EDM minimizes residual stresses, enhancing material strength and reliability^18,19^. Variations of EDM include sinking, dry, wire, and powder-mixed methods^20^. This diversity allows EDM to serve the requirements of both large-scale and small-scale manufacturing operations effectively.

The exceptional ductility, high hardness, superior toughness, viscosity, and hyperelastic behaviour of shape memory alloys pose significant challenges for traditional machining techniques. Achieving intricate geometries and precise dimensions using conventional methods becomes particularly difficult due to these properties. Applications of shape memory alloys span across diverse industries, including aerospace, medical devices, robotics, and construction materials. Products made from these alloys demand meticulous care and precise dimensional control due to their complex curvatures, intricate shapes, and fine features. Unlike traditional machining, electrical discharge machining (EDM) excels in producing complex geometries, micro-holes, and cavities without being constrained by the material’s hardness or toughness. This process utilizes electric spark erosion, a thermal technique that effectively operates independently of the material’s thermo-physical, chemical, or mechanical properties^21,22^. Discovered in the 1940s^23^, EDM has revolutionized machining by simplifying the processing of hard-to-cut materials. The process relies on thermoelectric energy generated by periodic electric discharges from a non-contact electrode, eroding the material from the workpiece submerged in a dielectric fluid^24–26^. During machining, spark generation produces electrons and positive ions, creating conductive pathways that form a high-temperature plasma channel. The rapid melting and evaporation in the high-temperature zone result in material removal from the workpiece^27–29^. Research on Fe-based shape memory alloys is less documented. Limited studies have explored their machining characteristics using advanced techniques like EDM, Wire-EDM, and LBM. Moreover, optimization methods like the Taguchi approach are more commonly applied, with fewer studies leveraging response surface methodology (RSM). Despite extensive SEM analyses on other SMAs, including NiTi, the surface features of Fe-based SMAs have received inadequate attention^3^.

Despite SMAs’ outstanding properties, their industrial adoption remains limited due to poor machinability using conventional methods. Recent research efforts have begun addressing these challenges. For instance, Chaurasia et al.^30^ investigated advanced discharge energy control strategies for NiTi SMAs to improve microstructural outcomes, while Dutta et al.^31^ demonstrated hybrid EDM-laser techniques for enhanced machining of complex bio-compatible SMA components. Notably, Ullah et al.^32^ highlighted the scarcity of machining data for Fe-based SMAs, identifying them as a promising but underexplored class of smart materials. Furthermore, Sana et al.^33^ and Farooq et al.^34^ proposed novel optimization frameworks (combining RSM and AI-based models) to predict and enhance EDM performance metrics for hard-to-machine alloys. These recent contributions underline the relevance and timeliness of our study, which uniquely focuses on Fe-based SMAs using Cu-W electrodes in an RSM-CCD framework, supported by SEM-based surface analysis^35^. Both studies^36,37^ enhance EDM performance for aerospace alloys (Ti6Al4V and Al6061) using cryogenically treated electrodes and modified dielectrics. The first study used Span-based non-ionic surfactants in kerosene with Al/graphite tools, improving MRR and surface quality. The second used deionized water with Al₂O₃ nanopowder and Span-20, achieving significant gains in MRR, surface finish, and energy efficiency with cryo-treated brass tools. Advanced modeling (ANN, NSGA-II) further optimized results. Both approaches address EDM limitations, offering sustainable, high-efficiency machining for hard-to-cut materials.

This study investigates the effects of electrical discharge machining (EDM) on the machinability and surface quality of iron-based shape memory alloys (SMAs) using Cu-Tungsten electrodes. Output responses, including workpiece wear (WOW) and tool electrode wear (WOTE), are examined in relation to process parameters such as gap voltage (GV), peak current (Ip), pulse off time (Toff), and pulse on time (Ton). Experimental design is conducted using response surface methodology (RSM) with a central composite design (CCD). The significance of machining inputs is analyzed through analysis of variance (ANOVA). Additionally, microstructural changes on the workpiece and electrode surfaces caused by EDM are studied using scanning electron microscopy (SEM). This research addresses key gaps by evaluating machining parameters’ influence on surface quality and wear.

## Materials and methods

**Fig. 1:**
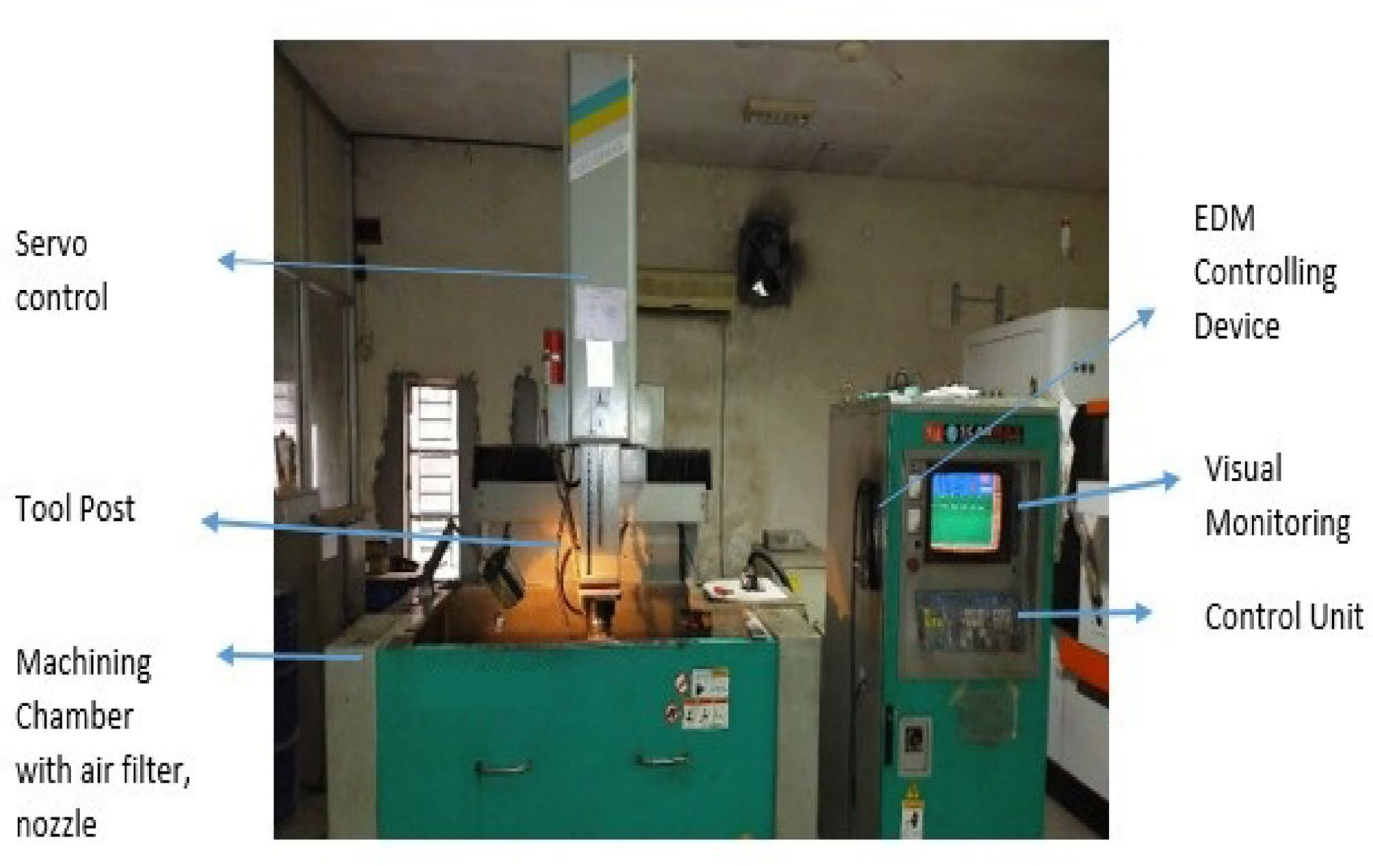
Diagram of CNC EDM set-up.

A shape memory alloy based on iron was used as the work sample for the EDM/ESCM processes. A trio of shape memory alloys are available. The bedrock of NiTi, Cu, and Fe… Figure 1 shows the results of the SEM and EDS characterizations performed on the Fe-based SMA prior to its application of the EDM technique. Although EDS (Figure-3) analysis mainly looked at Fe content, it also found additional elements including Cr, Ni, Mn, Si, etc. A Cu-Tungsten based electrode was used for electron beam machining (EDM) techniques. The round workpiece used in the EDM experiments has a diameter of 110 mm and a thickness of 15 mm. A copper-tungsten (Cu-W) electrode with a diameter of 10 mm serves as the tool. The key parameters considered for EDM include gap voltage (GV), peak current (Ip), pulse off time (Toff), and pulse on time (Ton). The key response factors evaluated under varying process settings are the wear of the workpiece (WOW) and the wear of the tool electrode (WOTE). Standard hydrocarbon-based EDM oil (commercial kerosene) was used as the dielectric fluid due to its wide availability and stable discharge performance. Although not biodegradable, it enabled consistent machining of Fe-based SMA in this study; however, future work will explore eco-friendly dielectrics to support green manufacturing goals.

The CNC EDM machine (Fig. 1) features servo control, tool post, chamber, nozzle, and monitor.

**Fig. 2:**
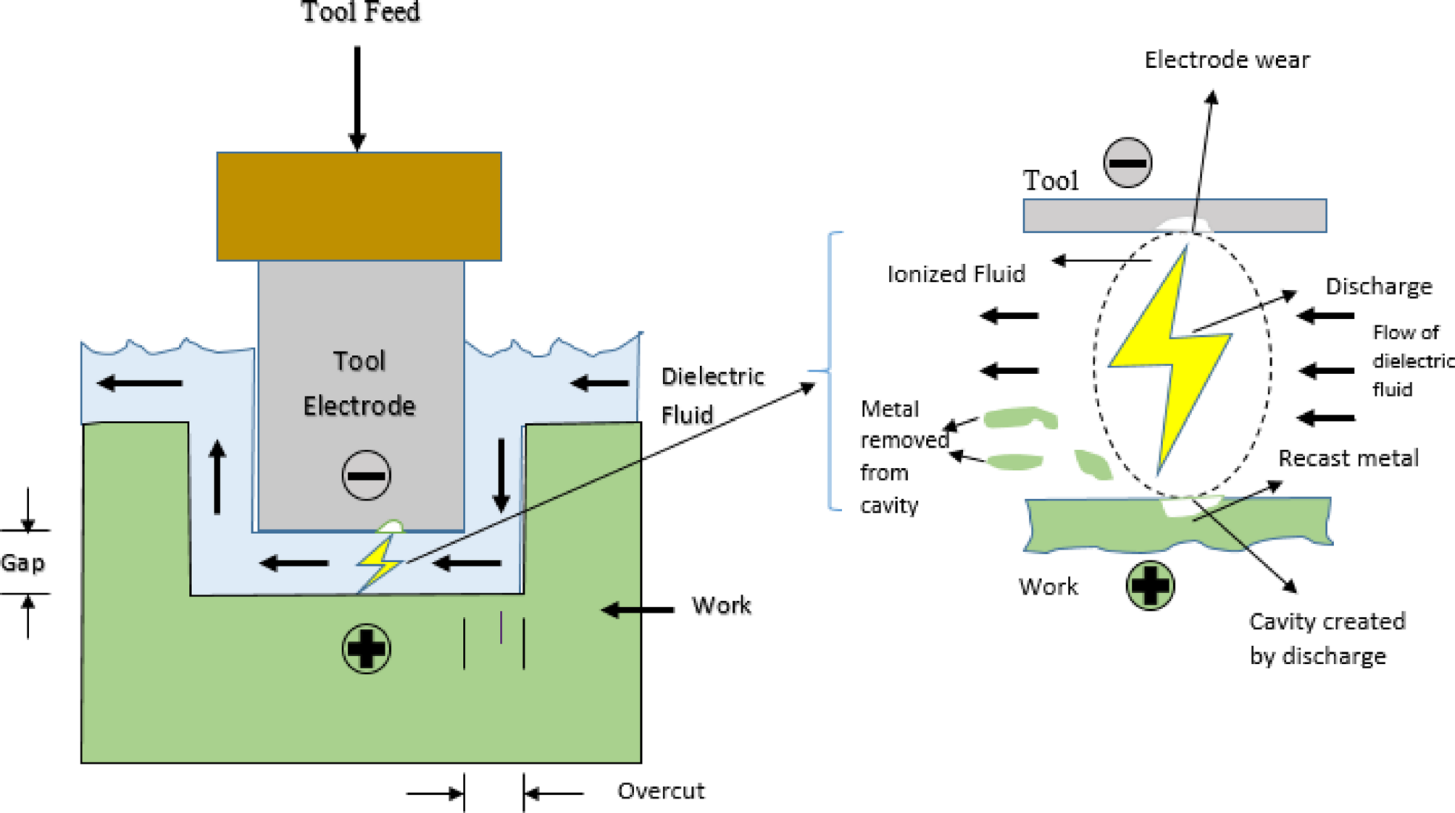
Machining zone and tool interface.

Figure 2 shows an enlarged view of the zone where the tool and workpiece interact. Many of spark’s characteristics have come to represent the idea of its inception. It shows dielectric fluid, tool feed, workpiece cavity development, electrode wear, and metal removal from the cavity. The symbols shown in Table 1 reflect Fe-based SMA.

**Table 1:** Configuration of Fe-based SMA.

Element	Fe	C	Si	Mn	*P*	S	Cr	Mo	Ni	Co	Cu	Ti
**Composition (%)**	66.00	0.044600	0.3140	1.580	0.04130	< 0.00500	18.200	2.0700	10.700	0.10400	0.3500	0.2250

**Fig. 3:**
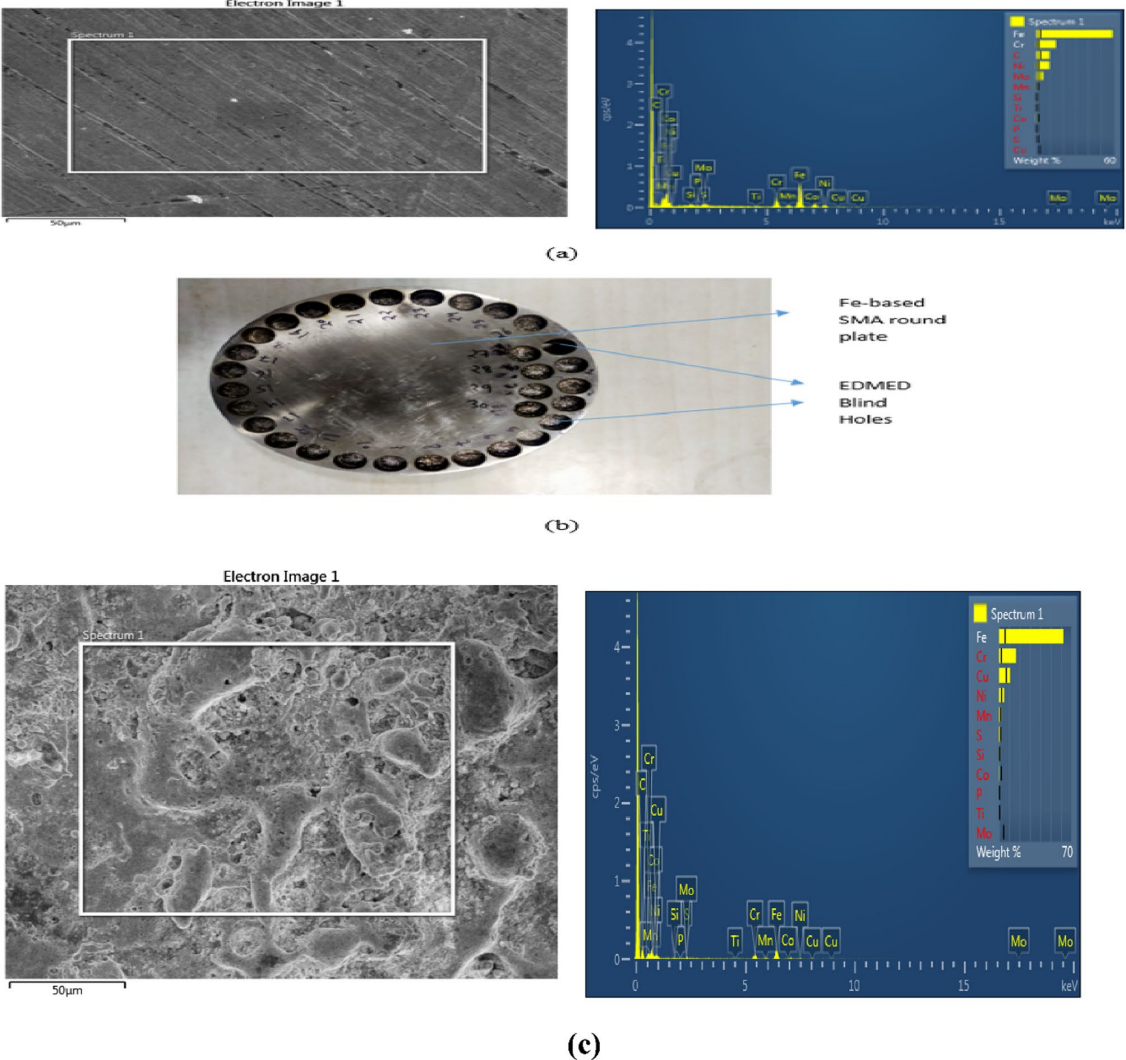
**(a)** EDS analysis spectrum **(b)** EDMed work sample disc **(c)** EDMed EDS analysis.

The experiments were conducted using a CNC EDM machine, as illustrated in Fig. 3. Key components of this machine include servo control, a tool post, a machining chamber, an air filter, a nozzle, an EDM regulator, a visual monitor, and a control unit. The experimental work material’s structure is reflected on these surfaces. SEM images reveal the work material’s outside layers in a parallel pattern. There is a much larger concentration of iron compared to the other elements. The main features of SMA that is based on iron are detailed in Table 2.

**Table 2:** Fe-based SMA properties.

Terms	Units	Values
Proof stress, UTS, Recovery stress	MPa	200 ~ 300, 680 ~ 1,000, 150 ~ 200
Ductility	%	16 ~ 30
Micro-hardness	Hv	190 ~ 220
Specific resistance	Ω·cm	100 ~ 130 × 10⁻⁶
Specific heat	cal/g·deg	0.13
Melting point	°C	1,320 ~ 1,350
Young’s modulus	GPa	170.0
Density (25 °C)	g/cm³	7.2 ~ 7.5 (7.454)
Thermal expansion (0 ~ 500 °C)	°C⁻¹	16.5 × 10⁻⁶
Shear modulus	GPa	65.0
Thermal conductivity	cal/cm·deg·sec	0.02
Recovery strain	%	2.5 ~ 4.5
Poisson ratio	--	0.359
Magnetic property	--	Paramagnetism

**Table 3:** Measures and process parameters considered.

Sr No.	Parameters	Symbol	L-1	L- 2	L- 3	L- 4	L- 5	Unit
1	Pulse on time	Ton	35	90	145	200	255	µs
2	Pulse off time	Toff	15	60	105	150	195	µs
3	Peak current	Ip	10	20	30	40	50	Amp
4	Gap voltage	GV	15	30	45	60	75	Volt

The study employed the Response Surface Method (RSM), a versatile approach for designing experiments and optimizing processes by analyzing the effects of independent variables on dependent responses. Initially introduced by Box and Wilson in 1951 during chemical experiments, RSM is widely used across various manufacturing processes, including extrusion, press working, riveting, cutting, milling, and spinning^38^. It also facilitates understanding the relationships between controlled machining parameters and response surfaces. Table 3 presents the optimal process parameter values, as well as their ranges. These were determined through extensive pilot tests and a comprehensive review of EDM process parameters from existing literature. Practical constraints and the availability of input on the EDM setup were also considered during this stage. Pilot tests evaluated the impact of individual parameters on process outputs, enabling the selection of appropriate input ranges for the main experiments. RSM was applied for optimizing and designing the primary experiments. This technique allowed for a detailed analysis of how machining parameters influenced the response variables. By focusing on the interactions and effects of these parameters, the study finalized the process conditions to achieve optimal performance outcomes. It is also used extensively in the medical and industrial fields. According to^39^, the following stages are included in the RSM strategy procedure:

- Developing a thorough set of studies to credibly and reliably evaluate the target response.
- Building a model to help with analysis and comprehension.
- Finding the best experimental configuration to get the target response value, whether it the highest or the lowest.
- Visualizing the individual and cumulative effects of process factors using two- and three-dimensional graphics.

It is possible to describe the response surface as: when all factors are regarded calculable.

Given that: z = produce response, we may write z as a function of g1, g2, g3,…, gn. The goal is to optimize the response variable, z. The n-th autonomous factor, also known as the control factor, is represented as g_n. The following equations often characterize the second-order surface interactions in RSM: The above equation may be paraphrased as: “z” is the yield response, and “gn” is the nth controller factor. α is the regression coefficient. The symbol ϋ represents an arbitrary mistake. The α coefficients, which are also called fractional regression coefficients, quantify the change in z that occurs when gn is varied by one unit while holding all other parameters constant. As part of the inferred surface z, the g_n_ variables include terms that are linear, squared, and cross-product. The Box-Wilson Central Composite Design (or simply a central composite design) is a popular choice for RSM. It incorporates center points and extra “star points” into a factorial or fractional factorial design in order to measure curvature. Rotatability in central composite design is ensured by choosing α based on factorial experimental runs and design qualities. The value of α is equal to the total of the factorials raised to the power of one divided by four. The function α = [2k]^(1/4) is used to find the α value in a complete factorial design.

On the other hand, a fractional factorial design of resolution V may likewise consist of the factorial part. A Central Composite Design (CCD) (Table-4) based experimental design that utilizes the RSM technique has been used to evaluate the experiments in order to get great findings. The tool wear rate (WOTE) was recorded in g·min⁻¹ and can be converted to volumetric wear using the density of the Cu-W electrode (15.8 g/cm³) to facilitate material-specific TWR calculation.

**Table 4:** Control log for main experimentation.

Std	Run	Pulse on time (µs)	Pulse off time (µs)	Peak current (A)	Gap voltage (V)	WOW (mm^3^/min)	WOTE (g/min)
16	1	200	150	40	60	65.17	6.25
25	2	145	105	30	45	53.6708	6.18
7	3	90	150	40	30	32.2037	6.2
19	4	145	15	30	45	51.1425	6.17
4	5	200	150	20	30	58.9324	6.25
21	6	145	105	10	45	53.8409	6.34
14	7	200	60	40	60	57.3967	6.62
3	8	90	150	20	30	33.5441	5.87
22	9	145	105	50	45	58.3567	6.72
29	10	145	105	30	45	49.1487	6.2
12	11	200	150	20	60	61.3987	6.15
2	12	200	60	20	30	59.8466	6.28
28	13	145	105	30	45	53.417	6.21
27	14	145	105	30	45	50.4928	6.24
1	15	90	60	20	30	38.8506	5.73
13	16	90	60	40	60	41.0346	5.94
24	17	145	105	30	75	48.9192	6.08
26	18	145	105	30	45	49.0527	6.18
15	19	90	150	40	60	35.6941	6.04
20	20	145	195	30	45	48.5225	6.15
18	21	255	105	30	45	56.1029	5.95
8	22	200	150	40	30	55.8123	6.25
6	23	200	60	40	30	61.9221	6.55
10	24	200	60	20	60	58.1698	6.4
17	25	35	105	30	45	11.3016	5.04
9	26	90	60	20	60	32.8435	5.65
11	27	90	150	20	60	33.7434	5.63
23	28	145	105	30	15	55.0535	6.22
30	29	145	105	30	45	53.2038	6.15
5	30	90	60	40	30	45.8057	5.89

## Results and discussions

The elements that determine the output are WOW and WOTE. For your reference, we’ve included the output replies’ ANOVA findings in Tables 5 and 6. According to the test findings, the WOW model has an F-value of 54.16 and the WOTE model has an F-value of 5129.69, both show that the model is highly significant. This extraordinarily large figure, arrived at via computations of the Model F value, is very improbable to have arisen as a result of noise. A model term is deemed important if its probability is higher than or equal to 0.0500. Any number higher than 0.0500 does not indicate statistical significance for the factor in question.

**Table 5:** ANOVA for WOW.

Source	Sum of squares	df	Mean square	F-value	*p*-value	
Model	4191.26	14	299.38	54.16	< 0.0001	significant
A-Pulse on time	3517.02	1	3517.02	636.22	< 0.0001	
B-Pulse off time	6.63	1	6.63	1.20	0.2908	
C-Peak current	22.54	1	22.54	4.08	0.0617	
D-Gap voltage	2.49	1	2.49	0.4510	0.5121	
AB	0.1101	1	0.1101	0.0199	0.8896	
AC	2.10	1	2.10	0.3807	0.5465	
AD	0.0315	1	0.0315	0.0057	0.9408	
BC	4.85	1	4.85	0.8781	0.3636	
BD	17.00	1	17.00	3.08	0.0999	
CD	5.56	1	5.56	1.01	0.3319	
A²	595.20	1	595.20	107.67	< 0.0001	
B²	10.74	1	10.74	1.94	0.1837	
C²	0.0961	1	0.0961	0.0174	0.8968	
D²	0.2091	1	0.2091	0.0378	0.8484	
**Residual**	82.92	15	5.53			
Lack of Fit	61.75	10	6.18	1.46	0.3547	not significant
Pure Error	21.17	5	4.23			
**Cor Total**	4274.18	29				
**Std. Dev.**	2.05			**R²**	0.9844	
**Mean**	48.82			**Adjusted R²**	0.9698	
**C.V. %**	4.20			**Predicted R²**	0.9356	
				**Adeq Precision**	35.5605	

A-Pulse on Time; B-Pulse off Time; C-Peak Current; D-Gap Voltage.

The model is considered excellent when there is no significant lack of fit (LOF). In the WOW scenario, the F-value for LOF is 1.46, and for the WOTE situation, it is 2.53, both of which are lower than the pure error. This suggests that the LOF is not statistically significant. In the WOW case, the likelihood of an F-value of this magnitude occurring due to random fluctuations is 35.47%, while in the WOTE case, the probability is 24.98%.

**Table 6:** ANOVA for WOTE.

Source	Sum of squares	df	Mean Square	F-value	*p*-value	
Model	0.0292	14	0.0021	5129.69	< 0.0001	Significant
A-Pulse on time	0.0146	1	0.0146	35761.26	< 0.0001	
B-Pulse off time	4315 × 10^−6^	1	4315 × 10^−6^	1.06	0.3197	
C-Peak current	0.0000	1	0.0000	30.70	< 0.0001	
D-Gap voltage	0.0002	1	0.0002	481.60	< 0.0001	
AB	9791 × 10^−6^	1	9791 × 10^−6^	2.40	0.1419	
AC	7735 × 10^−6^	1	7735 × 10^−6^	1.90	0.1884	
AD	0.0001	1	0.0001	307.41	< 0.0001	
BC	1440 × 10^−6^	1	1440 × 10^−6^	0.3536	0.5609	
BD	6233 × 10^−6^	1	6233 × 10^−6^	1.53	0.2351	
CD	1010 × 10^−7^	1	1010 × 10^−7^	0.0248	0.8770	
A²	0.0136	1	0.0136	33508.98	< 0.0001	
B²	4453 × 10^−7^	1	4453 × 10^−7^	0.1093	0.7455	
C²	6459 × 10^−6^	1	6459 × 10^−6^	1.59	0.2272	
D²	4573 × 10^−9^	1	4573 × 10^−9^	0.0011	0.9737	
**Residual**	6109 × 10^−5^	15	4073 × 10^−6^			
Lack of Fit	4832 × 10^−5^	10	4832 × 10^−6^	1.89	0.2498	Not significant
Pure Error	1278 × 10^−5^	5	2555 × 10^−6^			
**Cor Total**	0.0293	29				
**Std. Dev.**	0.0006			**R²**	0.9998	
**Mean**	0.0280			**Adjusted R²**	0.9996	
**C.V. %**	2.28			**Predicted R²**	0.9990	
				**Adeq Precision**	319.6085	

A-Pulse on Time; B-Pulse off Time; C-Peak Current; D-Gap Voltage.

**Fig. 4:**
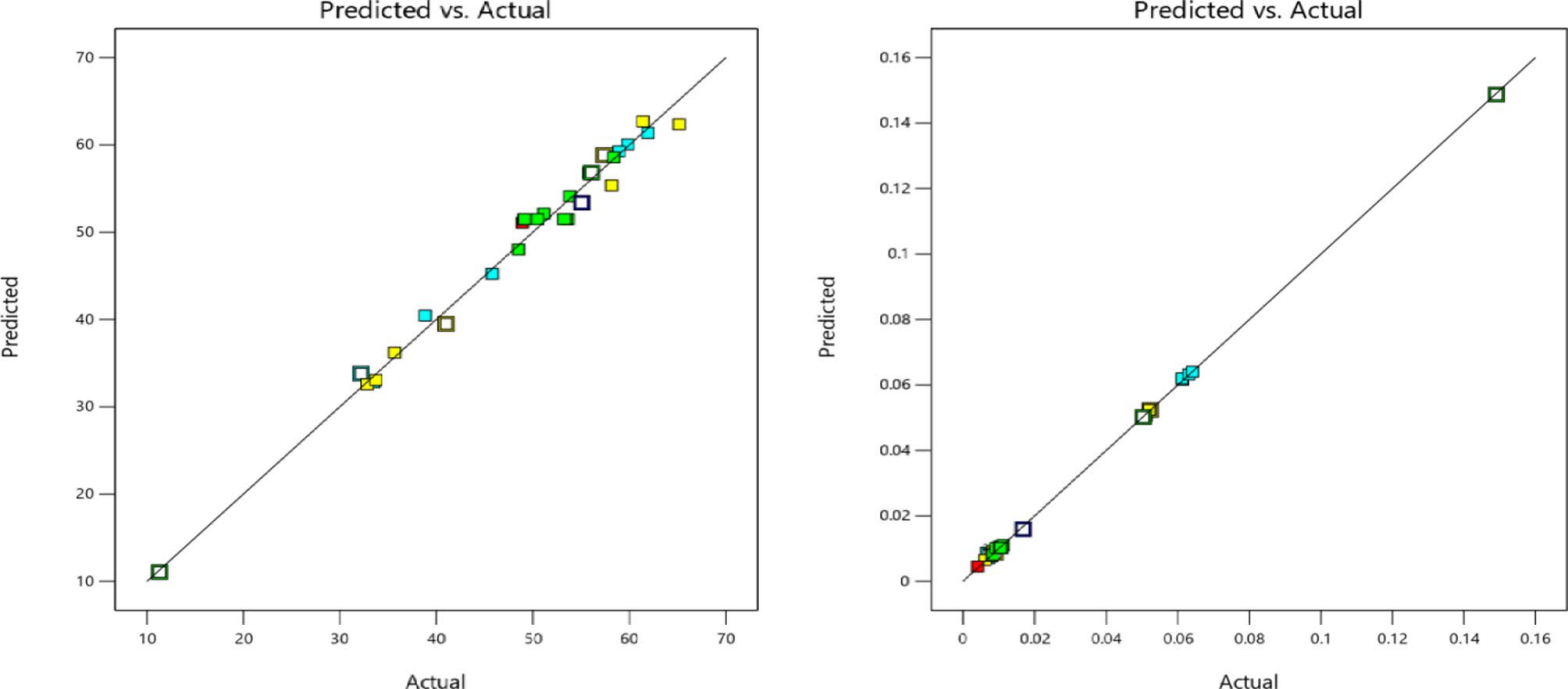
Predicted v/s Actual plot for (**a**) WOW (mm^3^/min) (**b**) WOTE (g/min).

**Fig. 5:**
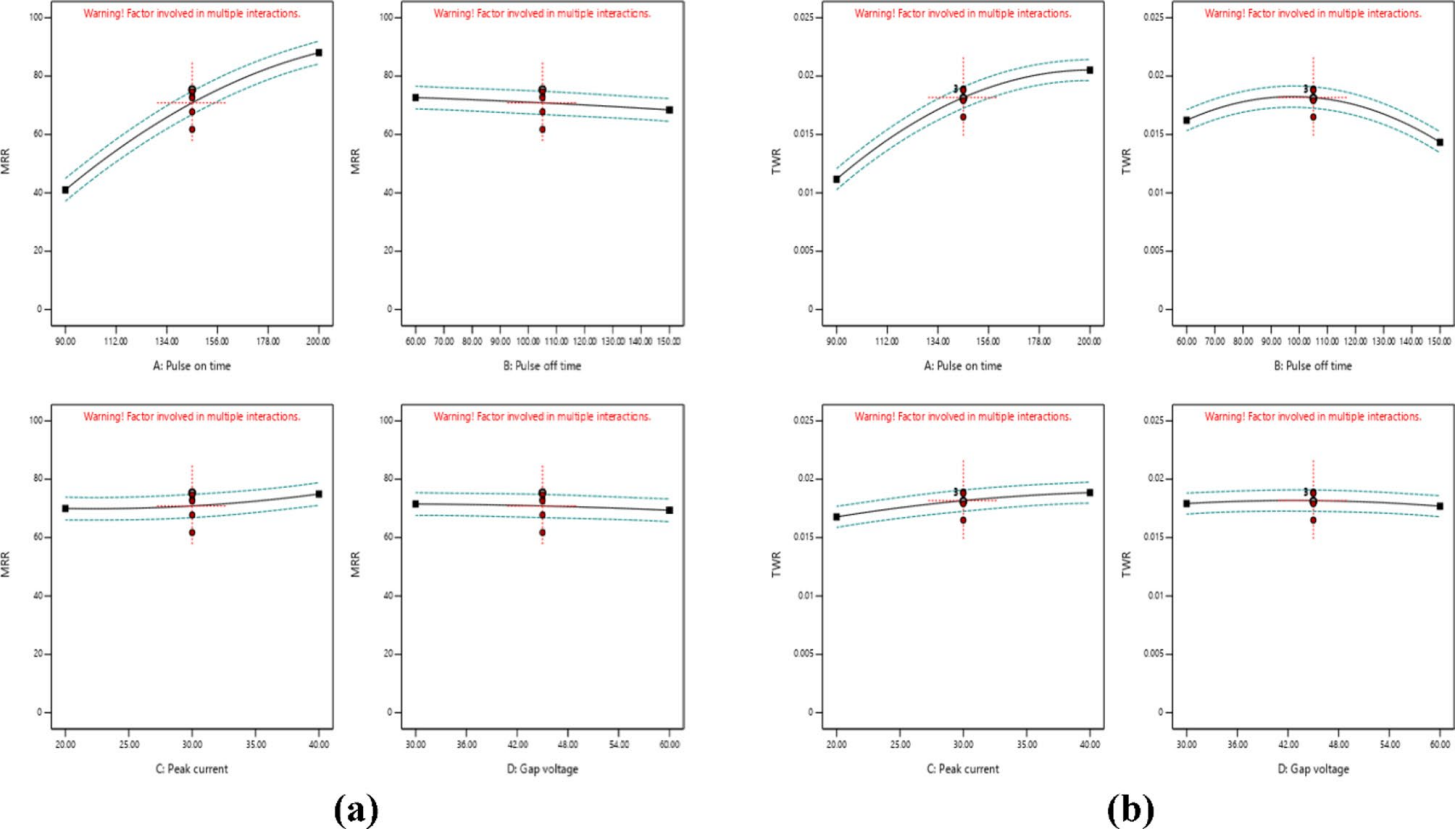
Single factor & EDM inputs on **(a)** MRR (WOW) (mm^3^/min) **(b)** TWR (WOTE) (g/min).

Figure 4 shows that the data flow in a straight line, which means that the model fits the data well. Ton, Toff, Ip, and GV impact WOW and WOTE in a two-dimensional representation in Fig. 5. Wow and WOTE both grow in proportion to an elevated heart rate. More sparks are produced when the pulse frequency increases due to the reduced lag time between pulses. The high sparking causes the tool and work to come into hot contact with one another. The surface becomes rougher as the melting and evaporation processes increase. Additional surface features such as fissures, craters, and debris formations are present on the work material. With an increasing pulse off time, WOW and WOTE decrease.

**Fig. 6:**
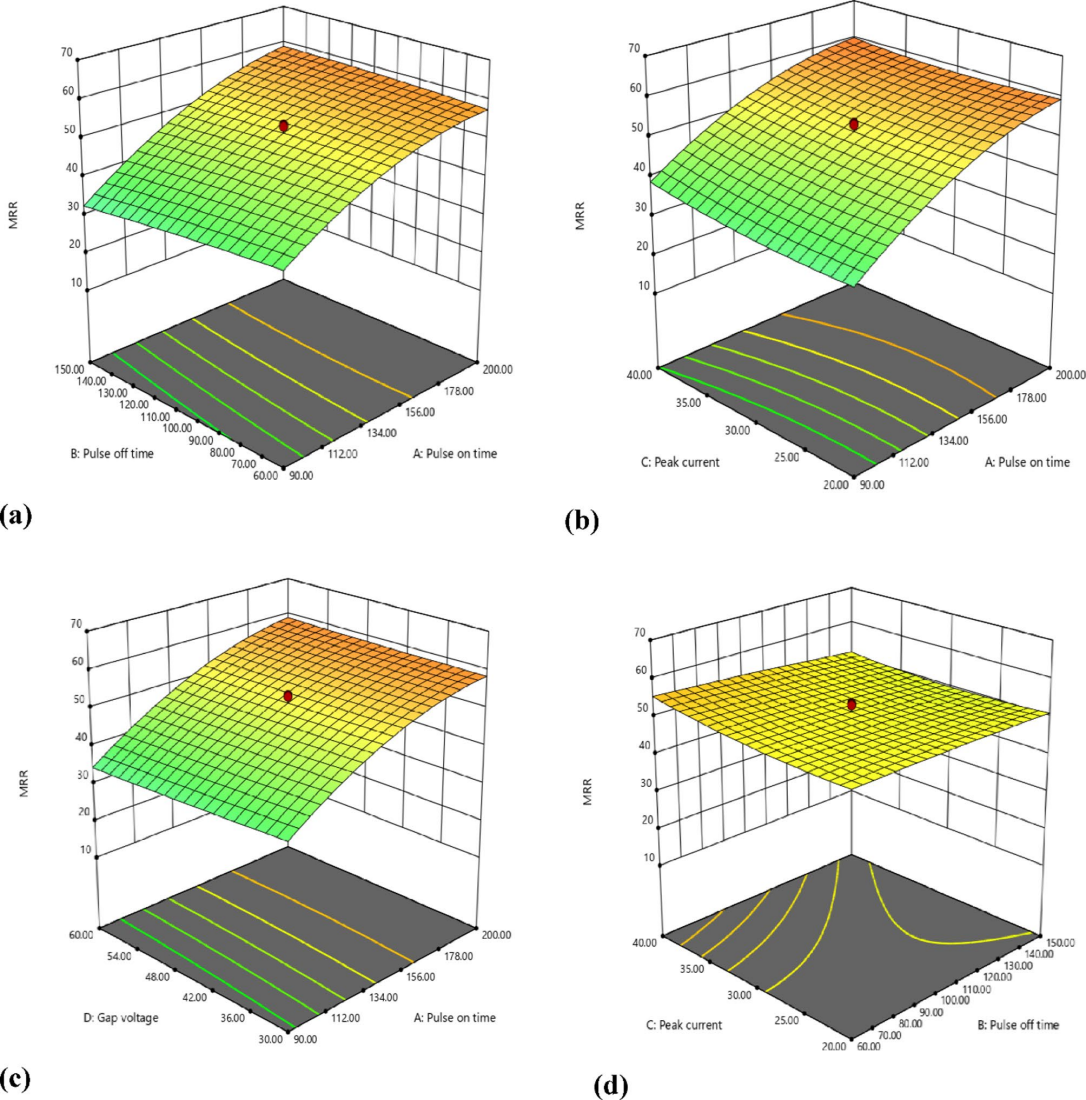
3D EDM inputs on MRR (WOW) (mm^3^/min).

Sparks are less likely to occur when the intervals between pulses are longer. The machining contact temperature decreases when sparks are decreased. Less material will melt and evaporate off the work surface because of the low temperature. A decrease in WOW and WOTE is caused by the scraping of less material off the surface. Higher current increases temperature. The high temperature has increased the amount of WOW and WOTE. A higher gap voltage is associated with a lower WOW and WOTE.

**Fig. 7:**
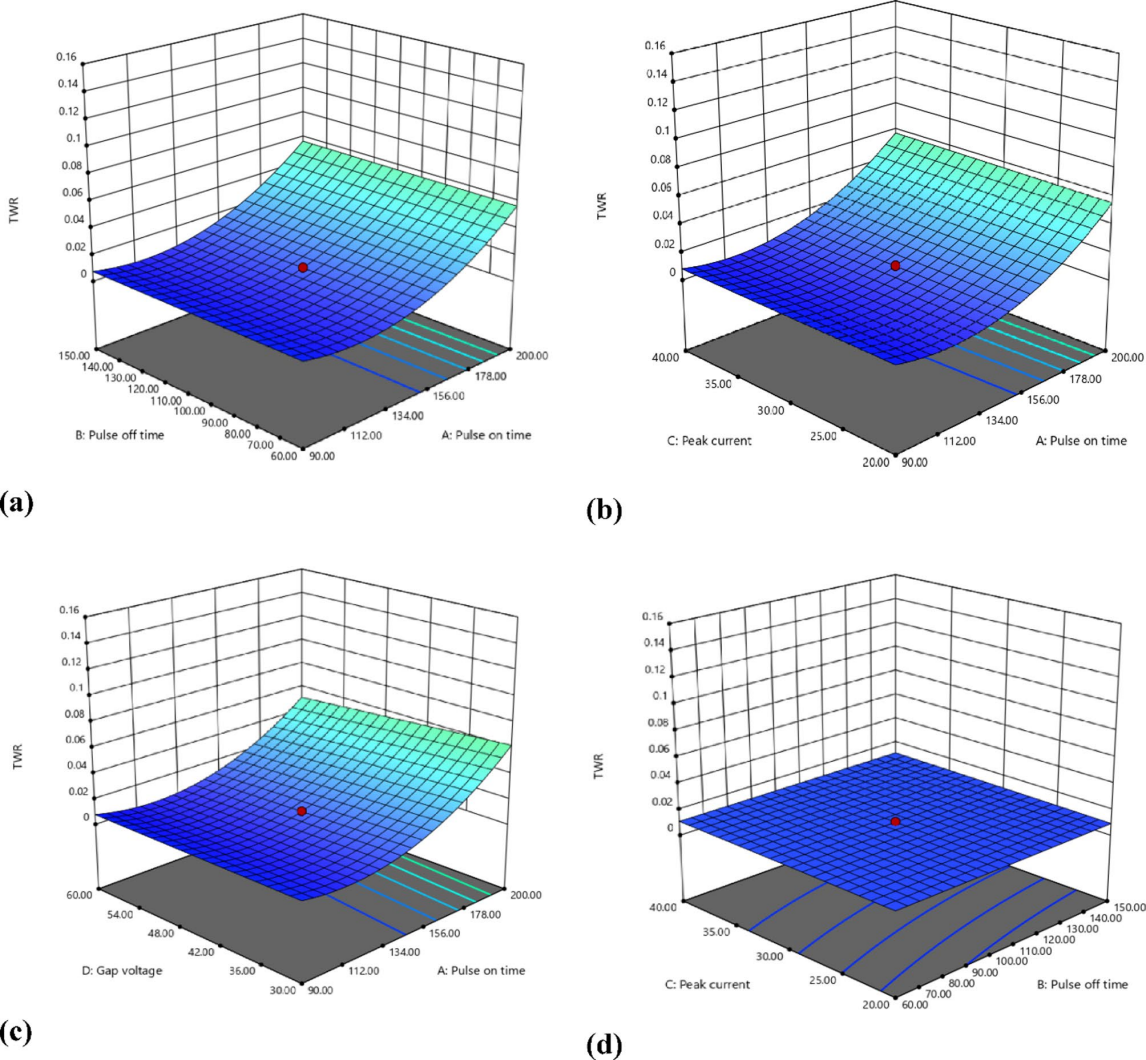
3D EDM inputs on TWR (WOTE) (g/min).

Figures 6 and 7 presents the three-dimensional response surface plots showing the interactive effects of key EDM process parameters (pulse on time (Ton), pulse off time (Toff), peak current (Ip), and gap voltage (GV)) on the Tool Wear Rate (WOTE) for the Fe-based SMA when machined with a Cu-W electrode. These 3D plots help visualize how combinations of input parameters influence the thermal energy distribution during machining, subsequently affecting the material removal from the tool. Notably, as Ton and Ip increase, the plots illustrate a rising trend in WOTE, attributed to higher discharge energy and prolonged spark duration, which accelerates tool erosion. In contrast, increasing Toff and GV appears to mitigate WOTE by allowing more cooling time and reducing spark intensity. These trends reinforce the thermal erosion mechanism dominant in EDM and support the optimization logic discussed later in the manuscript. The 3D surface plots shown in Figs. 6 and 7 were generated using the surf command in MATLAB for better visualization and convenience.

### EDMed microstructure: Cu-W on SMA

Debris globules, deep overlapping craters, RL development, and tiny fractures were all outcomes of the experimentally shown effects of the Ton and Toff intervals on surface integrity. Electrode machining (EDM) involves melting the workpiece material using discharges that produce heat, with the unwanted debris being removed using dielectric fluid.

**Fig. 8:**
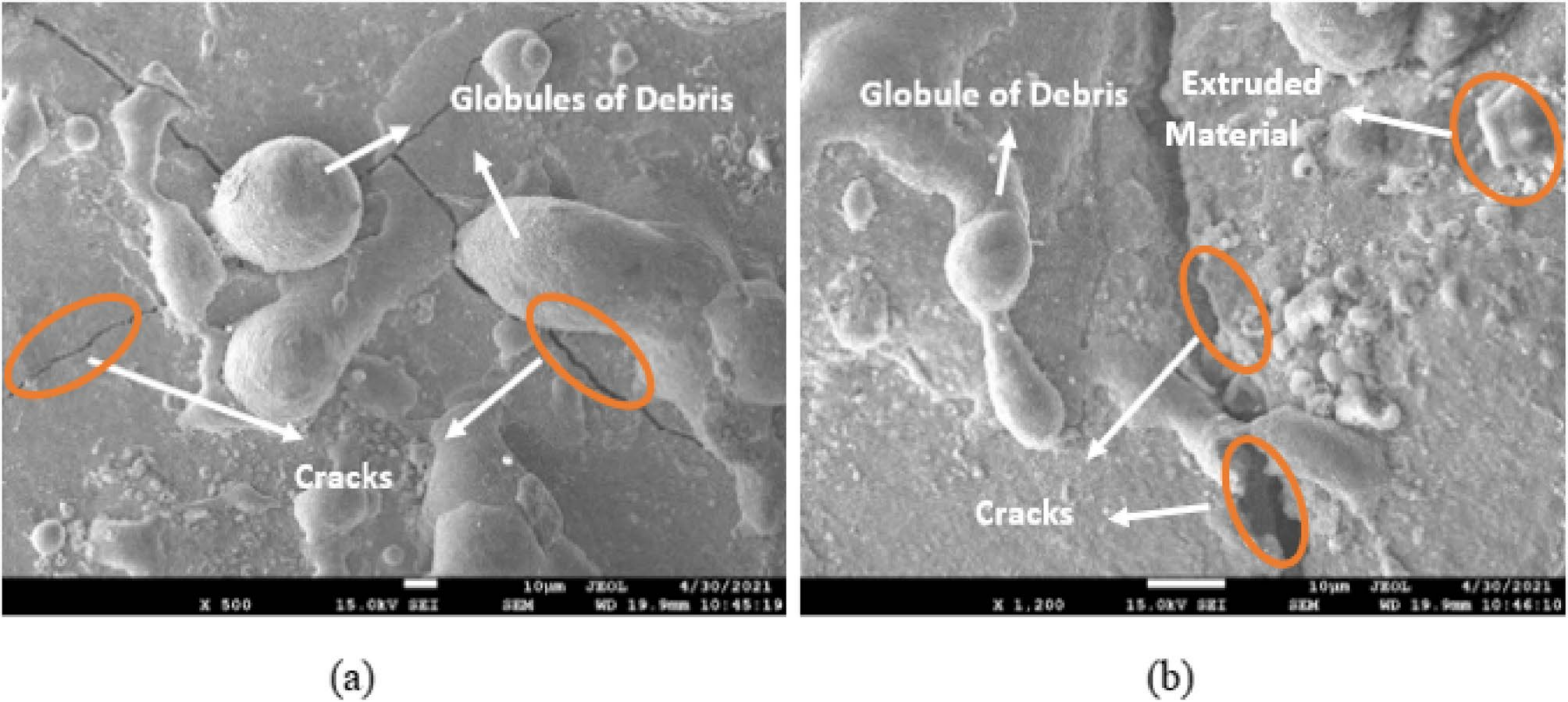
Fe-based SMA EDMed SEM Micrographs of Ton = 90 µs, Toff = 150 µs, Ip = 40 A, GV = 60 V (for EDMFe-CuW).

**Fig. 9:**
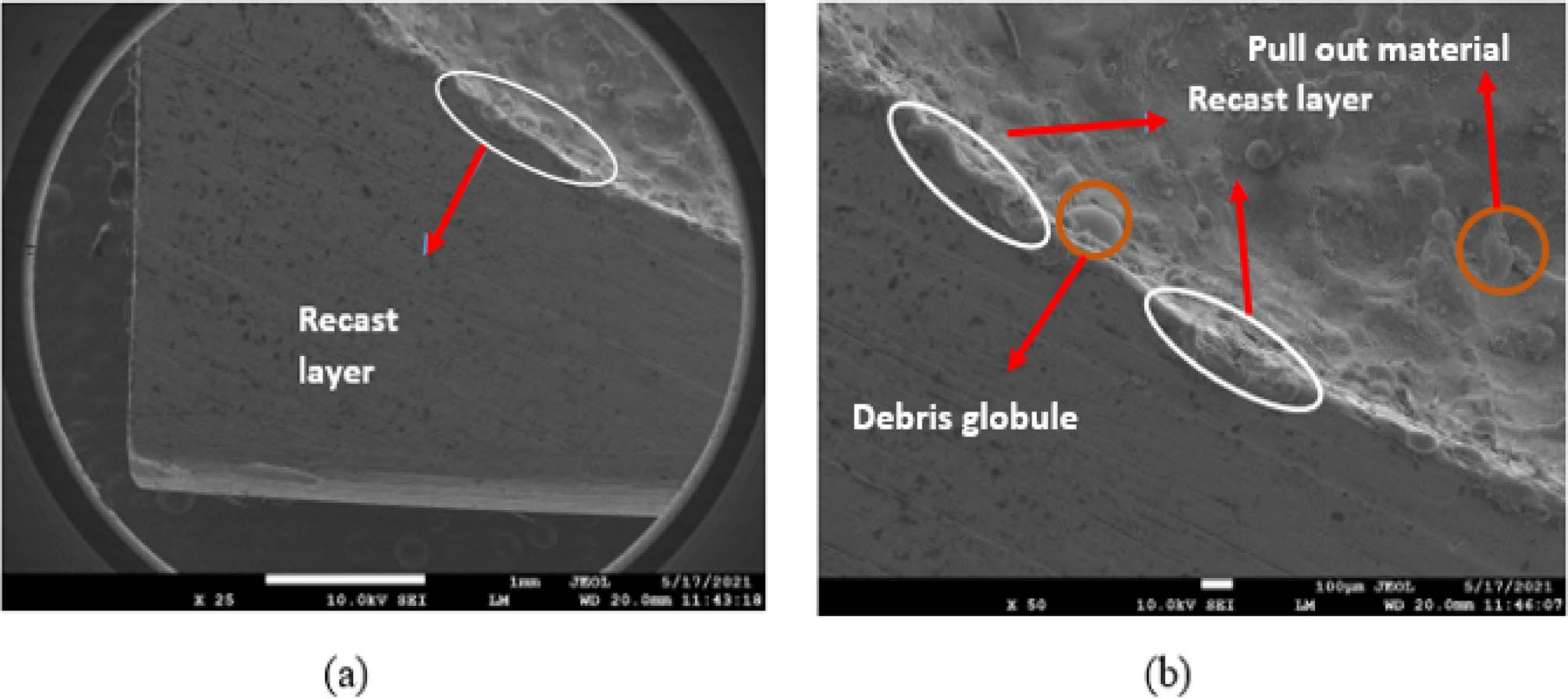
EDMed sample edges’ microstructure images of Ton = 90 µs, Toff = 150 µs, Ip = 40 A, GV = 60 V.

**Fig. 10:**
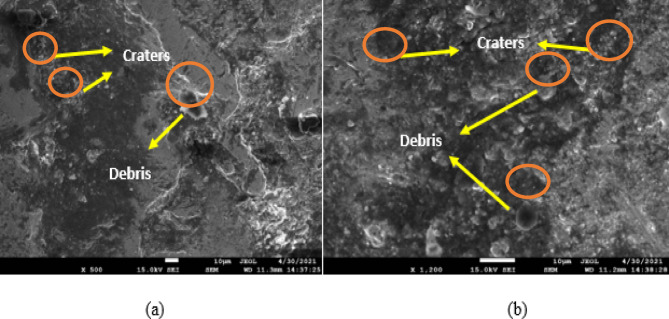
T_on_ parameter for Cu-W electrode = 90 µs, T_off_ = 150 µs, Ip = 40 A, GV = 60 V.

To test how different values of GV, Ip, Toff, and Ton affected the surface homogeneity of SMAs based on iron and copper, these variables were varied throughout the experiment. Scanning electron microscopy (SEM) was employed after electrochemical deposition microscopy (EDM) to evaluate the treated samples. The SEM has been used to examine the surface integrity of materials in this study. Surface morphology, which includes features like recast layers, heat-impacted zones, and microscopic scale fissures, is the most useful tool for describing the characteristics of a machined surface^40–43^. A number of process variables, including Ton, Toff, Ip, and GV, may change surface factors. Figures 8, 9 and 10 show the Scanning Electron Micrographs (SEM) taken of machined samples at various magnifications. Microcracks, craters, debris on surfaces. The effects of Ip and Ton on sample surfaces are substantial. Although qualitative SEM imaging confirmed recast layer development and surface cracking, quantitative evaluation of crack density and white layer thickness was beyond the current scope and will be addressed in future studies, particularly for assessing fatigue-critical applications.

Figure 8 depicts the SEM micrographs of the machined Fe-based SMA surface under the EDM condition of Ton = 90 µs, Toff = 150 µs, Ip = 40 A, and GV = 60 V. The surface exhibits overlapping craters, microcracks, and debris globules, indicating intense thermal cycles and rapid re-solidification. The craters suggest aggressive localized melting, while the microcracks stem from rapid quenching and thermal shock. Debris accumulation is apparent across the surface, forming re-solidified layers known as recast layers, which impact both surface integrity and dimensional accuracy. This figure validates the correlation between higher Ton and Ip values and surface degradation phenomena highlighting the significance of parameter control for applications demanding high surface quality.

Figure 9 shows the SEM microstructure of the EDMed sample edges under the same parameters as Fig. 8. This image focuses on the peripheral surface features, illustrating severe edge wear, deeper fissures, and thick recast deposition. These features reflect concentrated energy discharge near the sample boundaries due to electric field intensification, which enhances material removal but can compromise structural edges. The images provide visual evidence of edge instability, especially under prolonged Ton and high current, which is critical for part design involving sharp geometries or tight tolerances. Figure 10, although not requested, complements Figs. 8 and 9 by displaying the SEM microstructure of the Cu-W tool electrode. It reveals evidence of localized melting and wear pits, validating the WOTE values discussed in Fig. 7 and connecting the wear characteristics of the electrode to the observed machined surface morphology.

Increased debris from longer pulses (Fig. 8). Additionally, increasing the peak current and pulse-on time causes the work sample surfaces to become more etched, displaying more microcracks, craters, and debris globules. A greater pulse frequency increases the likelihood of a spark, which in turn increases the temperature^44–47^.

### Parametric optimization

#### Single response optimization

**Fig. 11:**
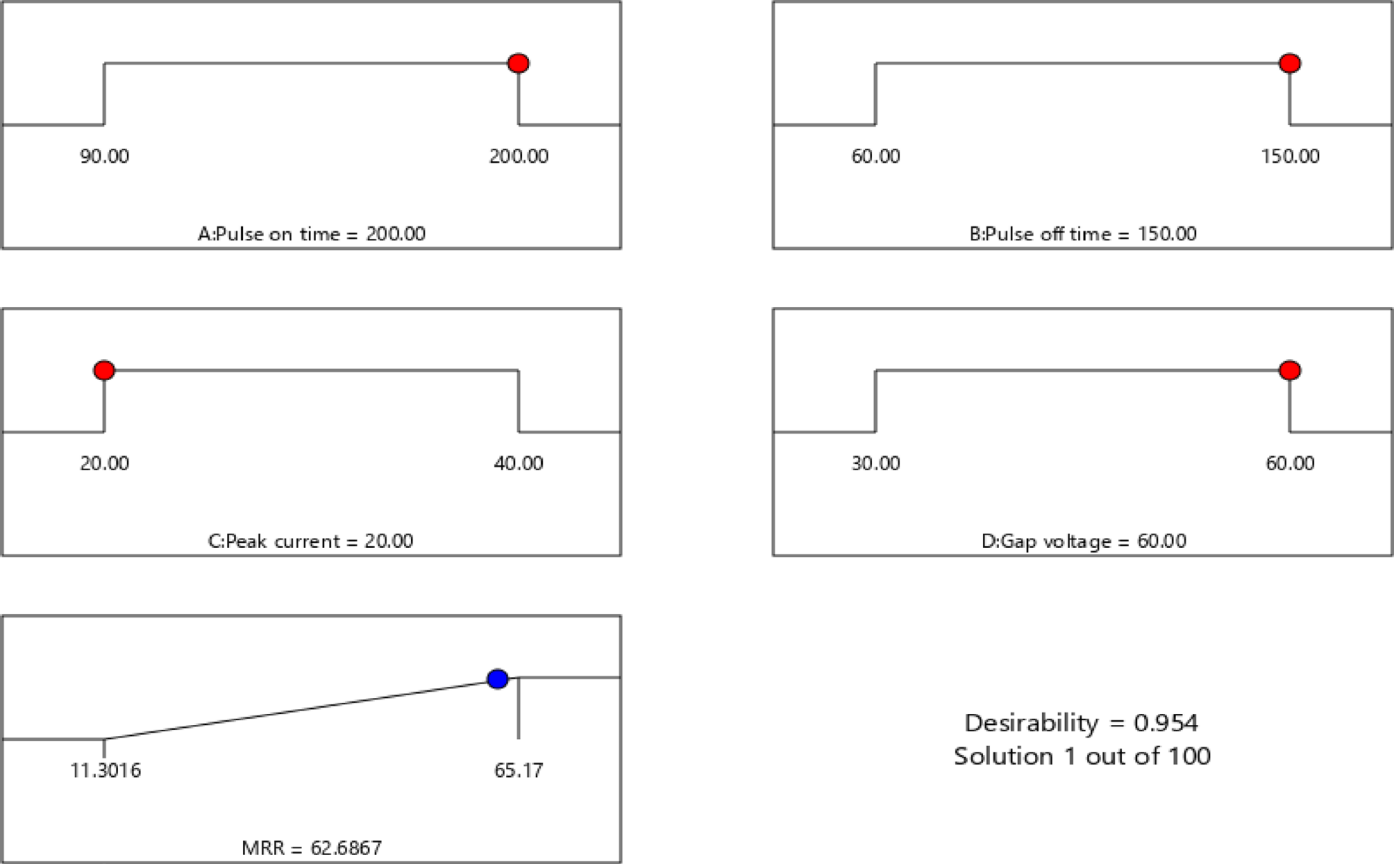
Desirability plot for MRR (WOW) at different optimized process parameter values (for EDMFe-CuW).

**Fig. 12:**
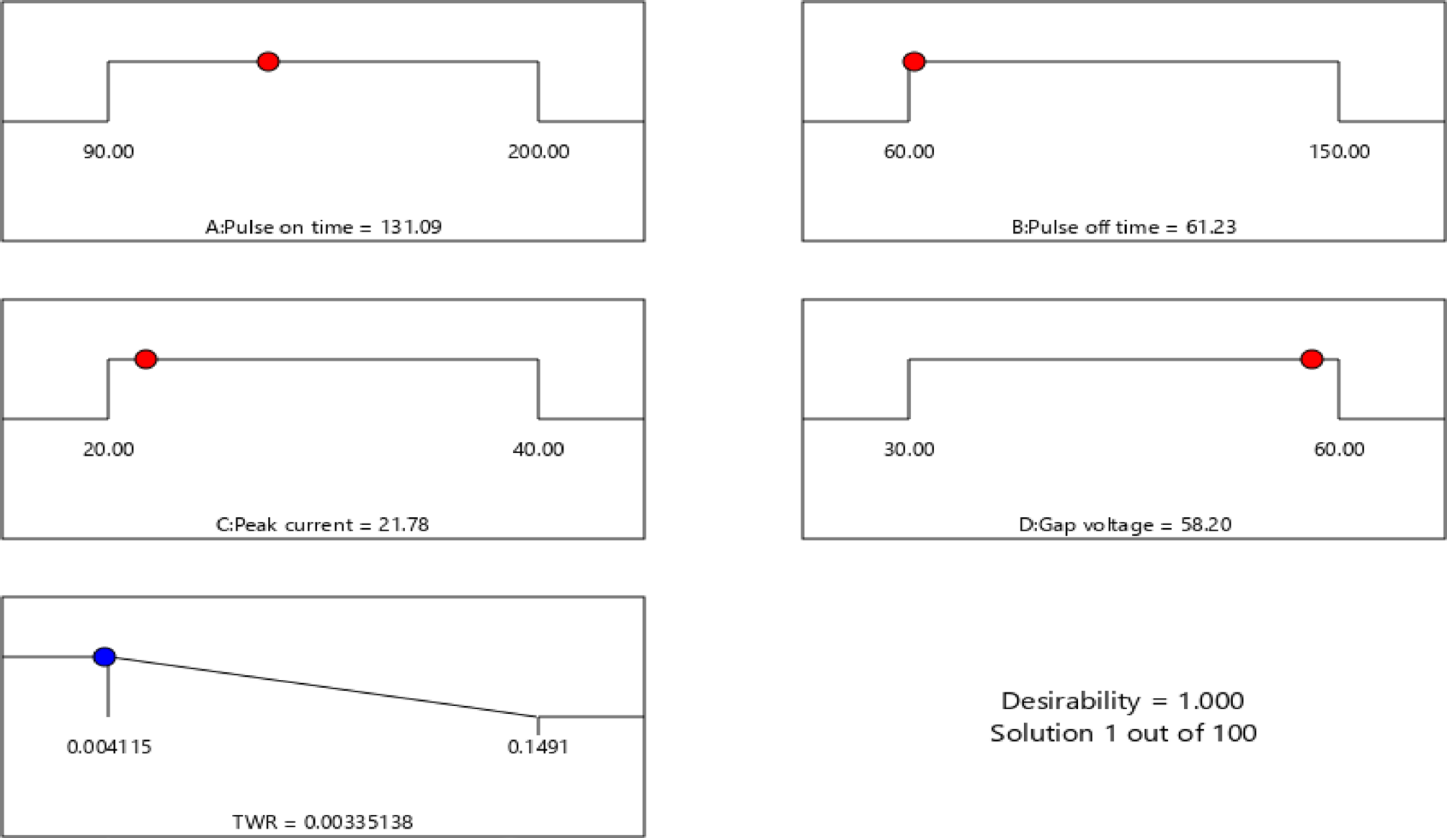
Desirability plot for TWR (WOTE) at different optimized process parameter values (for EDMFe-CuW).

The current study investigates the EDM technique and its response parameters, WOW and WOTE, which are inherently at odds with one another. It is not possible to get the largest MRR and the lowest TWR at the same time using various parameter combinations. Obtaining machining outcomes that may optimize many objectives simultaneously is critical for industrial applications. Both single- and multi-response optimization have made use of the desirability technique to get optimal outcomes^21,48–50^. Tables 7 and 8 show the results of the single- and multi-response optimizations, respectively, for the EDMFe-CuW example, utilizing the desirability technique. In the context of single answer optimization, Figs. 11 and 12 show the WOW and WOTE desirability approaches, respectively.

**Table 7:** Single-response optimization of machining responses (WOW & WOTE).

S. No.	Optimization	Response(s)	Optimized conditions				Predicted values	Confirmatory results	Best experimental results
			Pulse on time (µm)	Pulse off time (µm)	Peak current (A)	Gap voltage (V)			
1.	Desirability Approach	WOW (mm^3^/min)	**200**	**150**	**20**	**60**	62.6867	64.75	65.17
		WOTE (g/min)	**131.09**	**61.23**	**21.78**	**58.20**	0.003351	0.006178	0.0062

**Table 8:** Optimization of machining responses multi-response (WOW & WOTE).

S. No.	Optimization	Response(s)	Optimized conditions				Predicted values	Confirmatory results	Best experimental results
			Pulse on time (µm)	Pulse off time (µm)	Peak current (A)	Gap voltage (V)			
1.	Desirability Approach	WOW (mm^3^/min)	**90**	**60**	**30**	**20**	40.452	42.652	65.17
		WOTE (g/min)					0.007664	0.007856	0.0062

## Conclusions

Useful results were obtained from investigations into the EDM process, which explored the potential to enhance process efficiency by machining Fe-based SMA using a Copper-tungsten electrode:

- The WOW in the EDM of the analyzed SMAs, namely Fe-based SMAs, is significantly affected by all of the investigated input process factors. For the response metrics WOW and WOTE that were studied, the most important factors in processing Fe-based SMA in EDM are pulse on time and peak current.
- As the pulse on time rises, the WOW and WOTE soar. As the pulse frequency rises, the amount of sparks increases because the interval between pulse occurrences reduces. More surface melting and work surface evaporation means more WOW and WOTE.
- For EDMFe-CuW, the optimized WOW is 65.17 mm³/min with parameters: pulse on time = 200 µs, pulse off time = 150 µs, peak current = 40 A, gap voltage = 60 V. WOTE optimization achieved 0.0062 g/min using pulse on time = 90 µs, pulse off time = 150 µs, peak current = 20 A, and gap voltage = 60 V.
- SEM pictures showed that the workpiece and the electrode made of copper and tungsten had craters, holes, and microcracks on their surfaces. When Ton and Ip are high, the surface is completely removed. Scanning electron micrographs revealed the accumulation of debris on the surface of the workpiece and the electrode tool. When Ton and Ip are high, the spark energy at the workpiece-tool contact zone is high, which leads to debris production.
- When the workpiece is eroded, material re-solidifies on its surface, leading to the formation of a recast layer. This occurs due to factors such as increased spark generation, elevated temperatures, extended pulse width, and high peak current. The recast layer becomes visible on the surface of the workpiece, particularly along its edges, where the re-solidified material is exposed.
- The process parameters that yielded the best results when using the desirability approach were as follows: for WOW, Ton = 200 µs, Toff = 150 µs, Ip = 20 A, GV = 60; for WOTE, Ton = 131.09 µs, Toff = 61.23 µs, Ip = 21.78 A, GV = 58.20 V. 7. In the case of EDM Fe-CuW, the optimal combination of the process parameters for optimizing the multiple responses (WOW and WOTE) was: Ton = 90 µs, Toff = 60 µs, Ip = 30 A, and GV = 20 V. Predicted experimental values for WOW and WOTE using the desirability technique are 40.452 mm3/min and 0.007664 g/min, respectively.

Although this study optimized performance parameters like MRR and TWR, it did not include direct measurements of energy consumption or dielectric degradation. Future investigations will incorporate real-time current trace analysis and power factor monitoring to assess energy efficiency and sustainability trade-offs.

## Data Availability

The datasets generated and/or analysed during the current study are not publicly available because the dataset belongs to the ongoing project but are available from the corresponding author on reasonable request.
